# Classifying age from medial clavicle using a 30-year threshold: An image analysis based approach

**DOI:** 10.1371/journal.pone.0311262

**Published:** 2024-11-22

**Authors:** Nela Ivković, Željana Bašić, Ivan Jerković

**Affiliations:** University Department of Forensic Sciences, University of Split, Split, Croatia; University of Lahore - Raiwind Road Campus: The University of Lahore, PAKISTAN

## Abstract

This study aimed to develop image-analysis-based classification models for distinguishing individuals younger and older than 30 using the medial clavicle. We extracted 2D images of the medial clavicle from multi-slice computed tomography (MSCT) scans from Clinical Hospital Center Split (n = 204). A sample was divided into a training (164 images) and testing (40 images) dataset. The images were loaded into the Orange Data Mining 3.32.0., and transformed into vectors using the pre-trained neural network Painters: A model trained to predict painters from artwork images. We conducted Principal Components Analysis (PCA) to visualize regularities within data and reduce data dimensionality in classification. We employed three classifiers that provided >80% accuracy: Support Vector Machine (SVM), Logistic Regression (LR), and Neutral Network Identity SGD (NNI–SGD). We used 5-fold cross-validation (CV) to obtain optimal variables and performances and validated data on the independent test set, with a standard posterior probabilities (pp) threshold of 0.5 and 0.95. The explainability of the model was accessed visually by analyzing clusters and incorrectly classified images using anthropology field knowledge. Based on the PCA, clavicles clustered into categories under 30 and 40 years, between 40 and 55 years, and over 80 years. The overall accuracy with standard pp ranged from 82.5% to 92.5% for CV and 82.5% to 92.5% for the test set. The posterior probability of 0.95 provided classification accuracy up to 100% but with a lower proportion of images that could be classified. The study showed that image analysis based on a pre-trained deep neural network could contribute to distinguishing clavicles of individuals younger and older than 30.

## Introduction

Age estimation is an important identification factor in the cases of unknown skeletal remains, especially in criminal cases, mass graves victims from armed conflicts [1, 2], or natural disasters [3]. It is also crucial when dealing with living individuals, especially in legal and judicial proceedings due to illegal migrations, human trafficking, etc. [4]. There are several anthropological methods for age estimation of adults from skeletal elements, such as the morphological changes of the pubic symphysis [5–7], the auricular surface of the ilium [8], and the sternal part of the fourth rib [9, 10]. These methods are applicable for age estimation of adults on a broader age range, with different estimation errors in different age periods. The skull and the palatine sutures can be informative for age estimation, but with biological variations, estimation ranges can be relatively wide [11–15]. The medial clavicle is one of the most informative skeletal elements for distinguishing young and middle-aged adults because it fuses the last of all the other skeletal elements [16]. Analysis of the documented skeletal collections showed that the maximum age when epiphysis and diaphysis on the medial clavicle were not fused was 18 to 24 years, and the complete fusion was visible at the earliest of 19 years [5, 16–19]. Studies on computed tomography (CT) images of patients set the oldest age without ossification at 20–22 years, partial ossification at 15–36 years, and the youngest age with complete fusion at 20 [20–22]. The study on radiographs showed that the oldest person without fusion was 21–24 years old, partial fusion was seen at 16–30 years, and the youngest person with complete fusion was 18 years old [23, 24]. Generally, the specimen sources for such analysis can vary from dry bones [5, 18, 25], CT images [4], radiographs [4], and ultrasound [26] and can depend on the population analyzed. The advantages of CTs over radiographs (and dry bones) are thin layers, high resolution, and the possibility of creation of 3D images [27–31].

With some differences in methodologies, usually, five stages of ossification changes on the clavicle can be recognized: the ossification center is not ossified; the ossification center is ossified, but the cartilage is not; the cartilage is partly ossified; the cartilage is ossified, and the union line is visible; the cartilage is ossified, and the union is not visible [32]. Morphological traits can also be informative for age estimation, such as the morphology of the medial part (convex, concave, straight), relief (smooth, rough), porosity (not visible, visible), ossified nodules (not visible, visible), edge morphology (blunt, sharp elevated, exostosis, bony growth), etc. For example, an age group younger than 25 will exhibit no union of the diaphysis and epiphysis, or the beginning of the union, smooth surface with sharp edges; and the person above 50 will show a complete union, medial part porosity, bony nodules, exostosis, and blunt edges [33].

The disadvantage of the morphological methods is their subjectivity, dependence on the experience, and familiarity with the extent of changes in specific age groups. Using artificial intelligence, machine, and deep learning could be a promising alternative to the classical approach, as previous studies showed a potential for automatizing biological profiling based on images of skeletal structures, particularly those based on binary classification tasks. For example, such an approach could be employed to classify sex and age (to younger/older) with an age threshold of interest [34].

Although image analysis and [34] deep convulsion neural networks have overperformed traditional methods in many fields and for many tasks like the detection of objects [35], facial recognition [36], recognition of specific facial features [37], and head position [38], the application of such approach is still relatively rare in forensic anthropology [39].

Thus, this research aimed to estimate the age classification performance of the medial clavicle using computer image analysis and deep neural networks.

## Materials and methods

### Materials

We used 204 MSCT images of the thorax and abdomen from the adult patients imaged at the University Hospital Center Split, with layer thickness ≤ 1,5 mm. We excluded patients with trauma and pathology on the clavicle that could affect the analysis. The patients were imaged on the Siemens Somatom Definition AS 128 (Siemens AG Medical Solutions, Erlangen, Germany) and Philips Ingenuity CT (Philips, Cleveland, US). The images were reconstructed using a soft tissue convolution kernel.

#### Ethics statement

The research was approved by the Ethical committees of the Clinical Hospital Center Split (Class: 500-03/23-01/205; Reference number: 2181-147-01-06/LZ-23-02 from October 26, 2023) and the University Department of Forensic Sciences (Class: 2181-227-05-12-19-0006; Reference Number: 024-04/19-03/00007 from May 22, 2019). This was a retrospective study. The MSCT images of patients above legal age (18 years) were collected from November 2, 2023, to November 30, 2023. The data was completely anonymized in the Clinical Hospital Center Split, coded, and only information on sex and age were collected. The anonymized images were afterward stored in the virtual skeletal collection database of the University Department of Forensic Sciences. Considering that data was collected retrospectively, no informed consent was required.

### Methods

The images were analyzed using the software OsiriX MD (v.12.0, Switzerland). They were opened using a 3D volume reading tool (3D VRT), where the left clavicle was cut off from the rest of the body elements and was oriented in the medial plane to minimize effect of the image rotation. This software and selected tools for 3D visualization enables positioning and rotating elements considering standard radiological planes. After that, the image was opened with the 3D surface rendering tool (3D SRT), visualized on 250 or 300 pixels, and saved in TIFF format. In the program Gimp (v. 2.10.32, Germany), the images were loaded and cropped to a size of 700x700 px.

**Fig 1:**
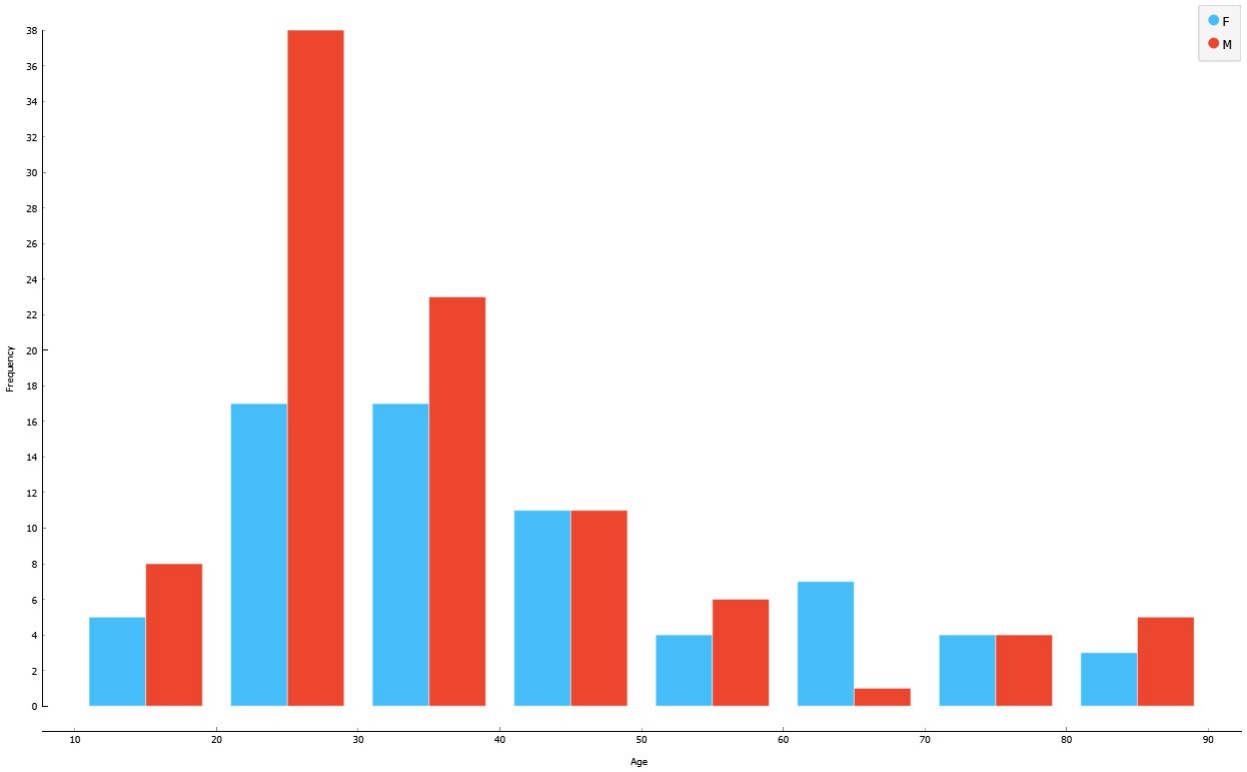
Sex and age distribution of the training dataset.

**Fig 2:**
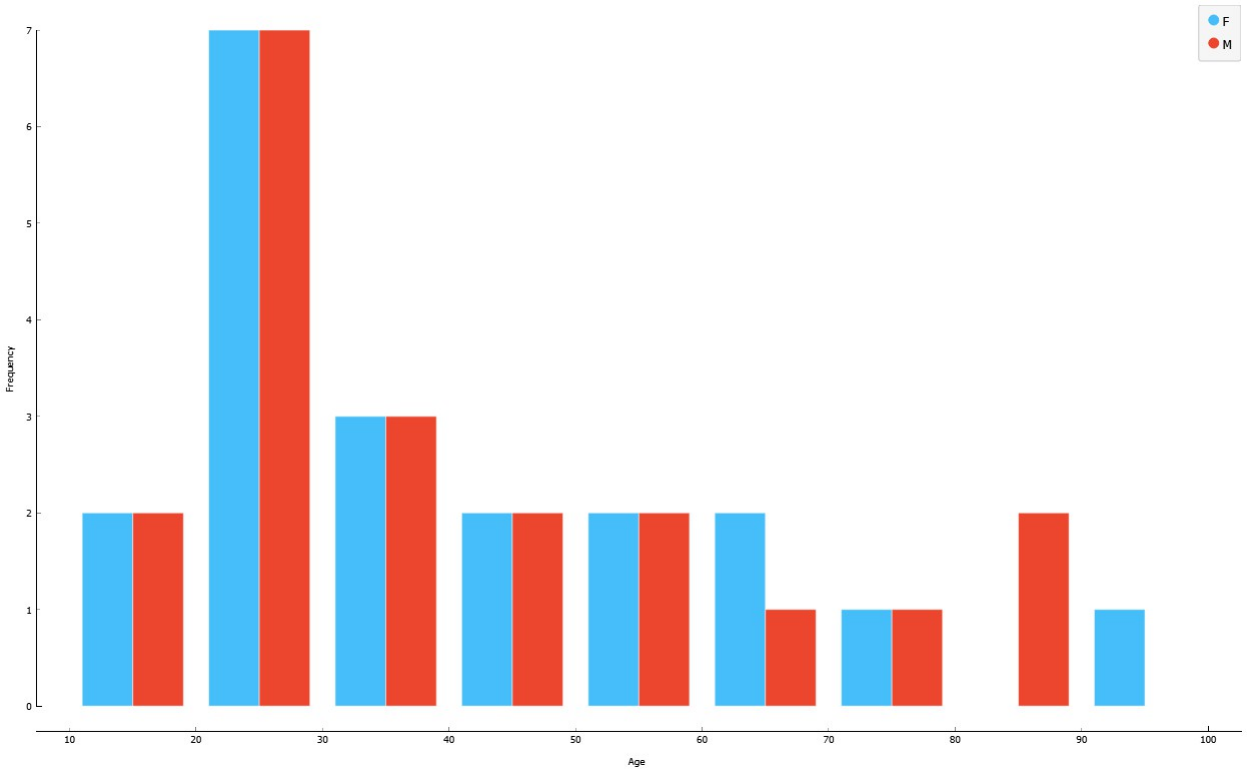
Sex and age distribution of the testing dataset.

The total of 204 images was split into the training set of 164 images of individuals, with a median age of 35 years, range of 18–90 years, and the testing set of 40 images of individuals, with a median age of 30 years, range of 18–92 years (Figs 1 and 2). The training set contained an equal number of males and females, while the test set was stratified by sex and age. To objectively grade and compare the efficiency of the algorithms and models, we used 5-fold cross-validation.

The images were imported into Orange Data Mining 3.32.0 (Ljubljana, Slovenija), a visual programming and data analysis software. Among many tools for data analysis, it enables the application of previously trained neuron networks for image analysis based on feature extraction [40].

We tested all available pre-trained networks available in the Image Embedding tool that is part of the Python Keras library: SqueezeNet [41], Inception v3 [42], VGG-16 [43], VGG-19 [43], Painters [44], DeepLoc [45]. The model that performed best was Painters, a neural network trained on 79433 images of 1584 artists [46]. Each image was transformed into 2047 features (variables) using this tool.

Exploratory data analysis was conducted using Principal Components Analysis (PCA) and the elbow method, which enabled the observation of regularities between data and the determination of whether the clavicles could be grouped by sex and age. For this study, we opted for age limit of 30 years, as it is considered upper limit when skeleton reaches maturity. Age estimation methods above this age differ from those based on epiphyseal fusion and focus on age-related degenerative changes [47, 48].

Data reduction was conducted using transformation on principal components. On cross-validation sample, we examined how many components gave the highest classification accuracy for the age limit of 30 years. The best result was obtained using eight components that explained 70% of the variance.

The following models were examined on cross-validation sample: Support vector machine—SVM [49], Neutral Network Identity SGD–NNI–SGD [50], Logistic Regression—LR [51], Stochastic Gradient Descent–SGD [52], Naive Bayes [53], k-Nearest Neighbors Algorithm–kNN [54], Tree [55], Gradient Boosting [56], Random Forest [57], and Adaptive Boosting, AdaBoost [58]. We used default settings for tuning parameters for those algorithms and chose those with accuracy above 80%. We tested their accuracy parameters on cross-validation and the independent test sample.

We tested the classification methods based on standard thresholds, where the posterior probability (pp) was 0.5, and a more conservative approach where classification was conducted only of specimens with pp higher than 0.95.

For the evaluation of classification models, we calculated true positive (TP), false positive (FP), true negative (TN), and false negative (FN) results, where younger than 30 years was considered a positive outcome (P). We also calculated accuracy, sensitivity, specificity, Positive Predictive Value (PPV), and Negative Predictive Value (NPV) [59].

To interpret patterns identified in exploratory data analysis (PCA) and detect the potential causes of misclassification, we visually inspected clustered and incorrectly classified images based on the anthropological traits [18, 33, 60, 61]. For this purpose, we directly visualized and analyzed images using the Orange tool Image Viewer.

## Results

### Exploratory data analysis

**Fig 3:**
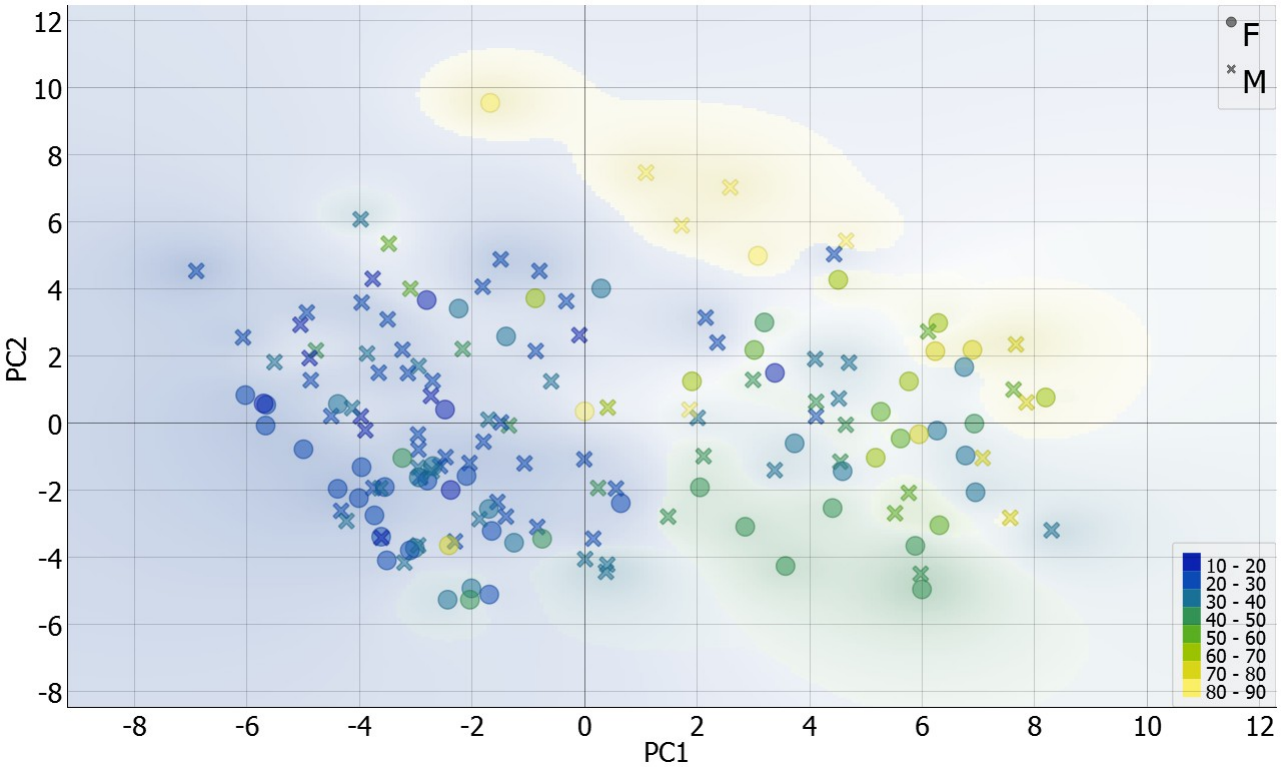
Distribution by sex and age on PC1 and PC2.

PCA analysis and elbow method showed that the optimal number of components that described the variance was three, and they explained 51% of the variance (PC1 explained 28.9%, PC2 explained 15.4%, and PC3 7.7%). The linear projection of the two components with labeled sex and age is shown in Fig 3. It is visible that the specific group categories clustered; most people younger than 40 were in the second and third quadrants, while most of the older adults were in the first and the fourth quadrants. No specific regularities were seen in sex distribution.

**Fig 4:**
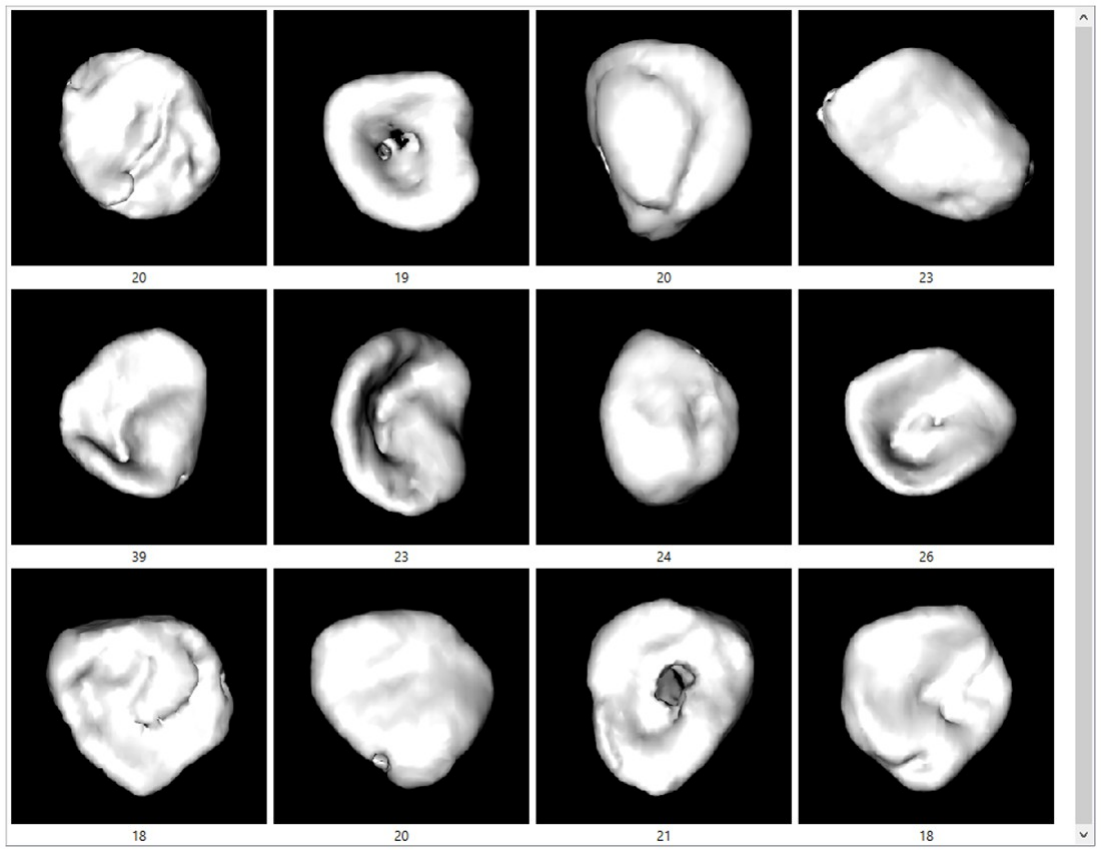
The examples of medial clavicles of younger persons located at the third and fourth quadrants of Fig 3 (shades of blue).

**Fig 5:**
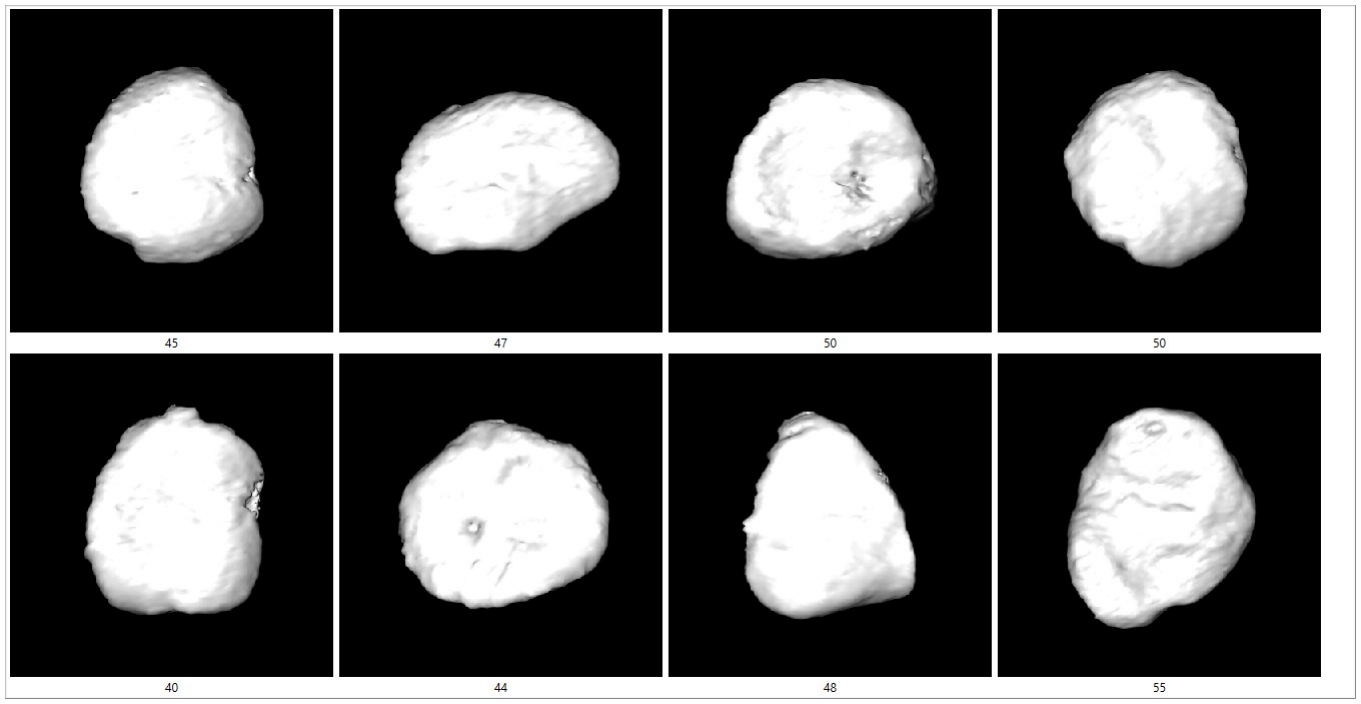
The examples of medial clavicles aged 40–55 years located in the second quadrant of Fig 3 (shades of green).

**Fig 6:**
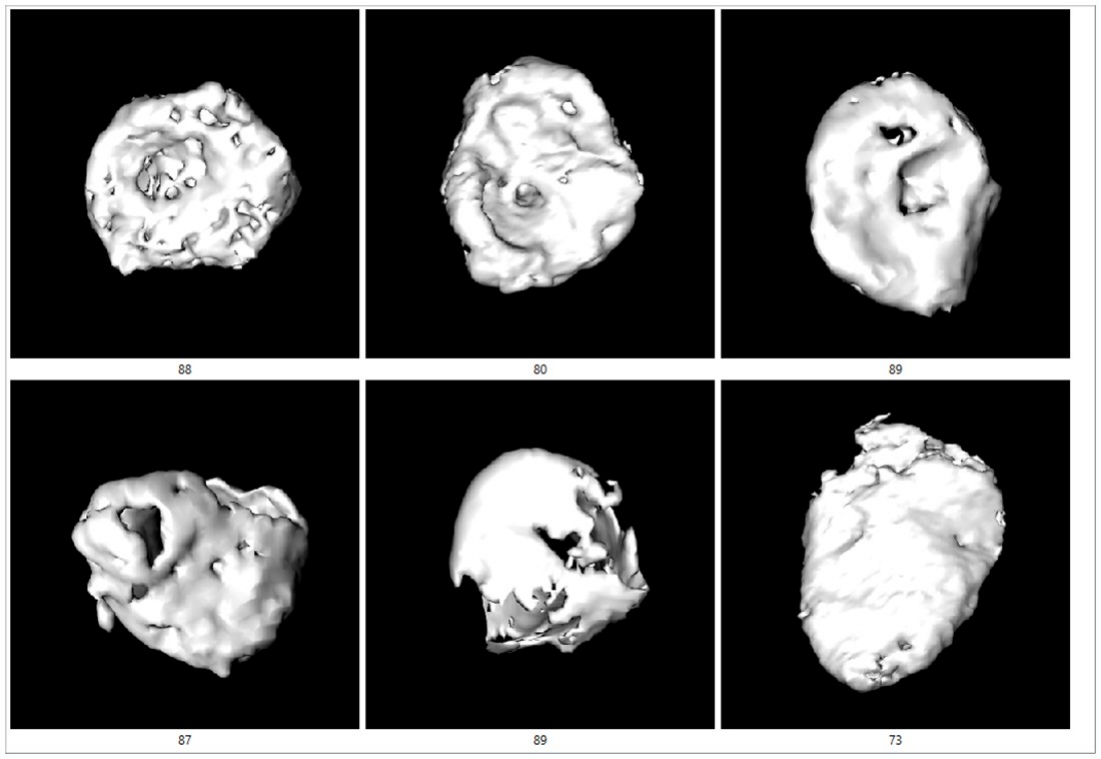
The examples of medial clavicles of persons older than 80 years on Fig 3 (shades of yellow).

In some images, the youngest age group showed incomplete epiphysis union or marks of a recent union, while most younger individuals demonstrated a billowed surface (Fig 4). Porosity and characteristic aging markers were common in individuals aged 40–55 (Fig 5), while more pronounced degenerative changes were typical in individuals older than 80 (Fig 6).

### Classification models for 30-year threshold and standard posterior probability

A total of 2047 features were reduced to eight principal components, giving the highest accuracy range of 0.677 to 0.823 (depending on the classifier used). The total cross-validation accuracy of classification algorithms ranged from 67.7% for Ada Boost to 82.3% for the SVM. Besides SVM, we included the remaining two algorithms whose accuracy was above 80% (LR and NNI-SGD, with 81.1%) for further analysis.

The model’s efficiency was further analyzed for chosen models using the standard pp of 0.5. Among the classifiers selected, the highest cross-validation accuracy was achieved with SVM, which also had the best balance between PPV and NPV. The other two algorithms had the same classification accuracy (Table 1).

**Table 1:** The classification performance of models on the cross-validation sample with pp = 0.5.

Model	Accuracy (%)	Sensitivity (%)	Specificity (%)	PPV (%)	NPV (%)
SVM	82.32	76.81	86.32	80.30	83.67
LR	81.10	75.36	85.26	78.79	82.65
NNI—SGD	81.10	76.81	84.21	77.94	83.33

Most incorrectly classified persons were in the age category 20–39 years, around the border values that were in this study set to 30 years. The number of incorrectly classified persons was smaller in the age category below 20 years, and the lowest number of incorrectly classified persons was in persons older than 50 years.

**Fig 7:**
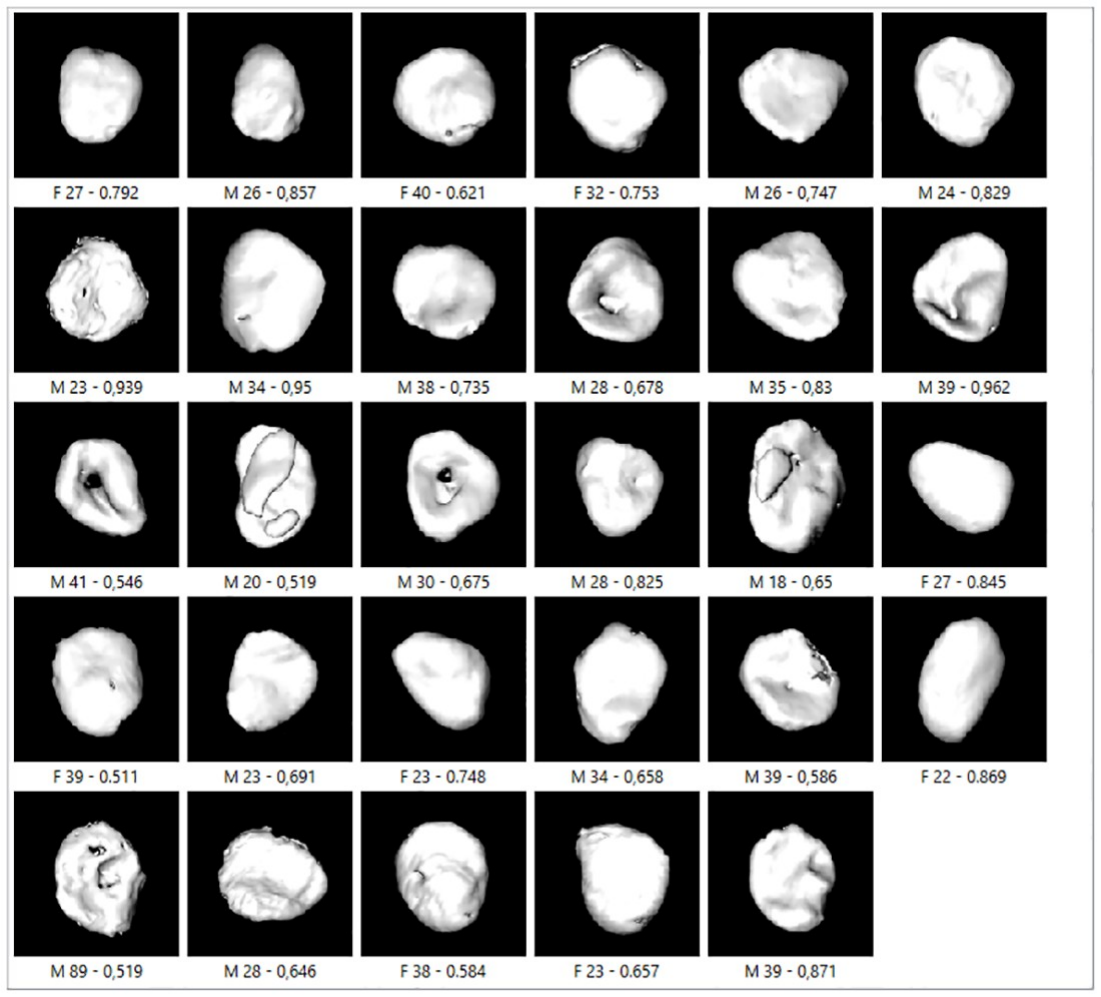
SVM–incorrectly classified clavicles on cross-validation sample with pp = 0.5.

**Fig 8:**
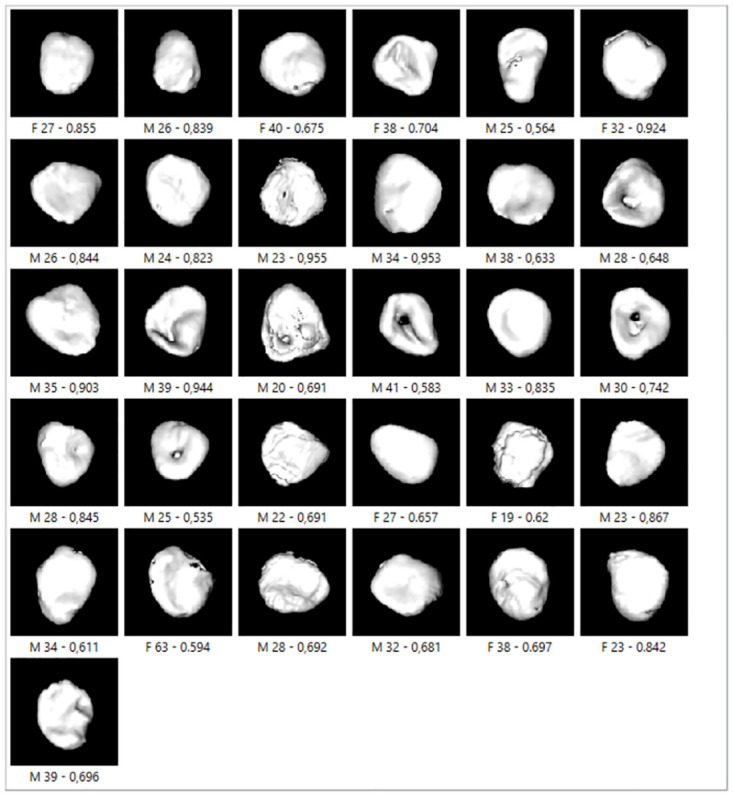
LR—incorrectly classified clavicles on cross-validation sample with pp = 0.5.

**Fig 9:**
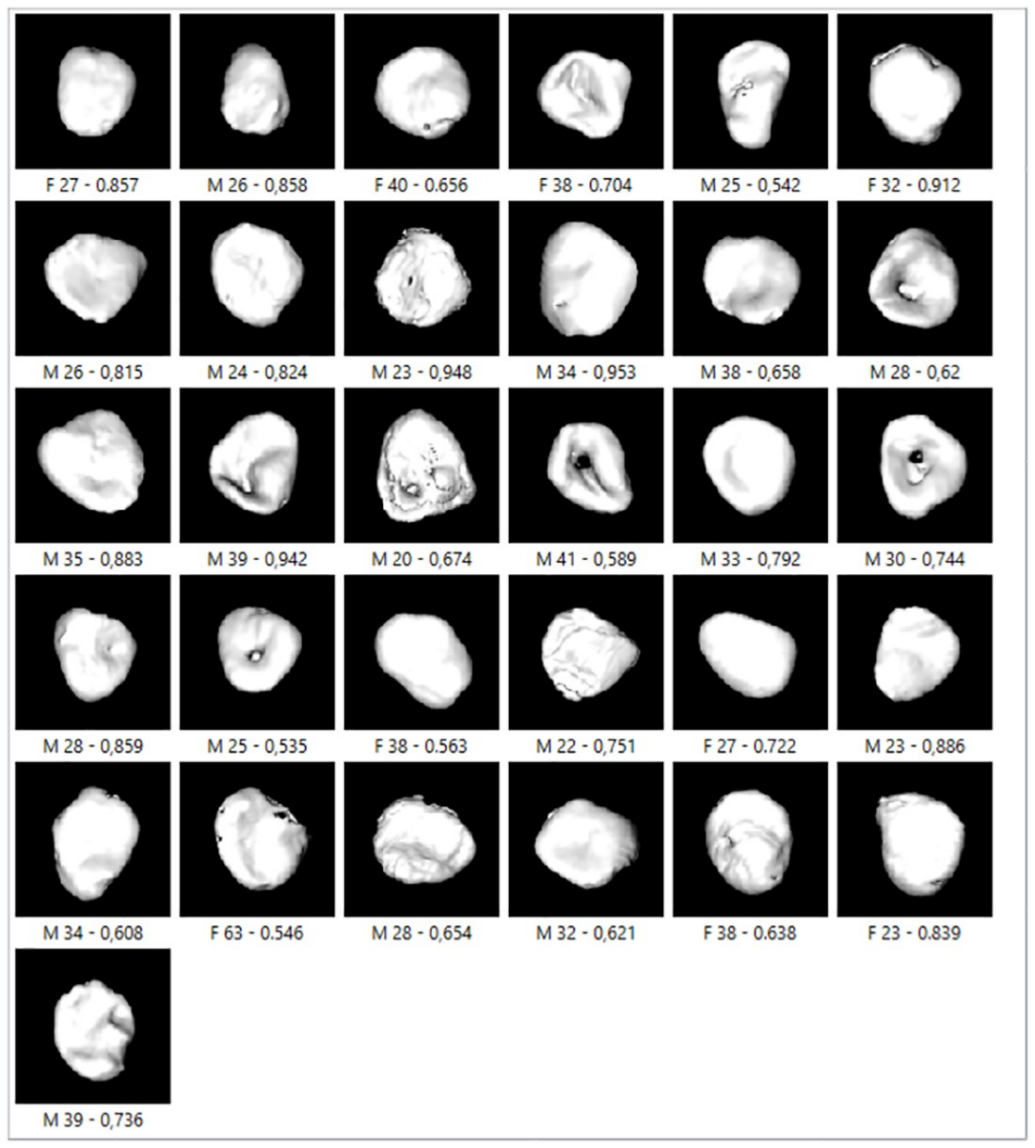
NNI—SGD—incorrectly classified clavicles on cross-validation sample with pp = 0.5.

Figs 7–9 show incorrectly classified medial clavicles and sex and age for all three classifiers. There were some males from older age groups whose medial clavicle resembled younger bones, for example, visible nodules and bone concavity that contoured the surface with shadow resembling unfused diaphysis; see male aged 89, in Fig 7. The opposite example was a male 23 years old (Figs 7–9); his bone showed porosity and roughness, which are more common in older age. The unfused diaphysis was visible for a woman aged 19 (Fig 8), but the edges were rough, which could be the source of the wrong classification. A male, aged 20 (Figs 7–9), had visible union marks, but the edges were slightly porous and rough; in two men, aged 30 and 41 (Figs 7–9) and one woman, aged 63 (Figs 8 and 9), bone perforations, leading to misclassification, were visible.

The total accuracy of classification algorithms on the test sample ranged from 82.5% for SVM to 92.5% for LR, while the classification accuracy for NNI—SGD was 90% (Table 2). In all cases, PPV and NPV were above 90%.

**Table 2:** Performance of the classification models on test sample (n = 40) with pp = 0.5.

Model	Accuracy (%)	Sensitivity (%)	Specificity (%)	PPV (%)	NPV (%)
**SVM**	82.50	75.00	90.00	88.24	78.26
**LR**	92.50	90.00	95.00	94.74	90.48
**NNI—SGD**	90.00	90.00	90.00	90.00	90.00

**Fig 10:**
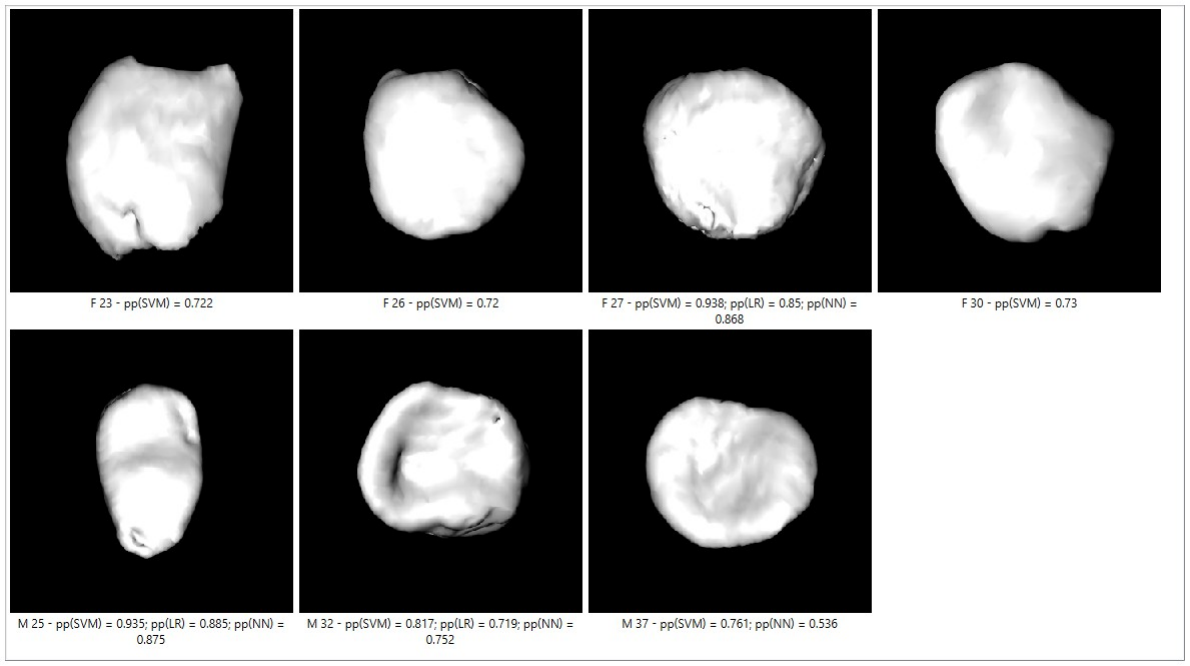
Incorrectly classified clavicles in test samples with SVM, LR, and NNI–SGD.

Fig 10 shows incorrectly classified clavicles on the test sample, including the data of sex and age of the person, and pp. SVM had seven incorrectly classified specimens, LR three, and NNI—SGD four. Interestingly, in most cases, these were the bones of the same person. For example, two clavicles of males (ages 25 and 32) and one clavicle of a female (27 years) were incorrectly classified with all algorithms, with relatively high pp. NNI—SGD and SVM incorrectly classified one male aged 37, with pp for NNI—SGD of 0.536. In SVM, pp was higher, and it classified incorrectly three female clavicles (23, 26, and 30 years old), with pp 0.72 and 0.73. When analyzing those images, it is visible that misclassification probably occurred because a male (25 years) and a female (27 years) had visible nodules, and a male (32 years) had a convex surface with expressed shadow. Two females (26 and 30 years old) had a flat surface, and one male (37 years) and one female (23 years) had a visible rough surface with irregular edges. All the described features were not consistent with their age.

### Classification models for a 30-year threshold using a posterior probability of 0.95

Classifiers’ accuracy was also tested using a posterior probability of 0.95. Using LR and NNI—SGD 41/164 (25%) clavicles were classified, while the SVM classified 8/164 (5.49%). The highest classification accuracy of 97.56% and the best balance between PPV and NPV was achieved using NNI—SGD (Table 3). LR performed slightly worse, with accuracy of 95.35%, while the lowest accuracy had SVM (85.71%).

**Table 3:** Classification performance of models on cross-validation sample with pp = 0.95.

Model	% of classified (n, %)	Accuracy (%)	Sensitivity (%)	Specificity (%)	PPV (%)	NPV (%)
**SVM**	8 (5.49)	88.89	100.00	80.00	80.00	100.00
**LR**	41 (25.00)	95.35	92.31	96.67	92.31	96.67
**NNI—SGD**	41 (25.00)	97.56	100.00	96.55	92.31	100.00

**Fig 11:**
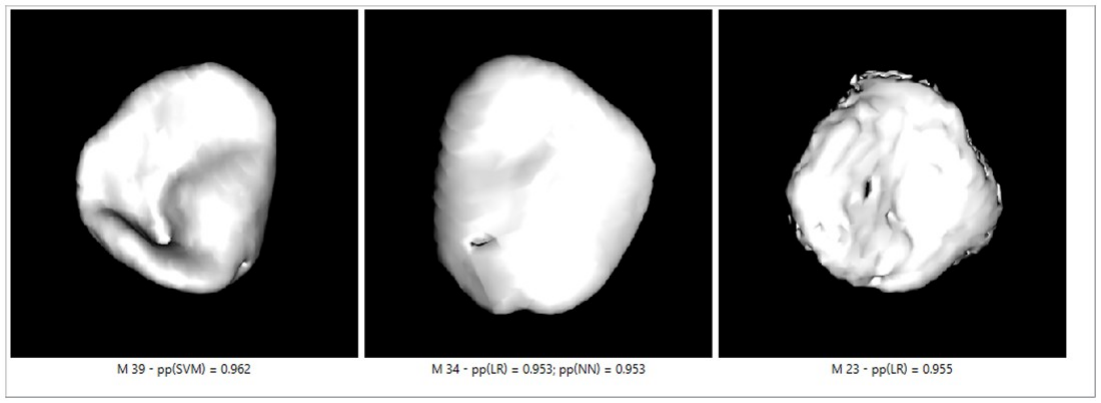
SVM, LR, and NNI–SGD—incorrectly classified clavicles on cross-validation sample with pp = 0.95.

Fig 11 shows the misclassified bones for pp = 0.95 and three different classifiers. For example, a 39-year-old male had a medial clavicle with a visible nodule and bone concavity. This created a contoured shadow on the bone surface resembling the epiphysis and diaphysis union marks, which categorized him in a lower age category. On the clavicle of a 23-year-old male, a surface with epiphysis and diaphysis union was visible. Still, the bone edges were irregular, the bone surface was porous, and this person was classified as older. The same bone was incorrectly categorized with two classifiers (male, 34 years old), and the reason for misclassification is probably the confusion between two different markers—smooth surface but containing a hole.

The proportion of images classified with pp 0.95 was higher in LR 15/40 (37.5%) than in NNI—SGD 13/40 (32.5%), while SVM could correctly classify only one person (Table 4).

**Table 4:** Classification performance of the models on test sample (n = 40) with pp = 0.95.

Model	% of classified (n, %)	Accuracy (%)	Sensitivity (%)	Specificity (%)	PPV (%)	NPV (%)
**SVM**	1 (2.5)	/	/	/	/	/
**LR**	15 (37.5)	100.00	100.00	100.00	100.00	100.00
**NNI—SGD**	13 (32.5)	100.00	100.00	100.00	100.00	100.00

## Discussion

This study showed that image analysis of the medial clavicle could distinguish between younger and older adults with relatively high accuracy, depending on the classification model and posterior probability. While the approach with a standard threshold provided accuracies between 82.65 and 83.67% for CV and between 82.5 and 92.5% for the test sample, the more conservative threshold of 0.95 provided CV accuracies of 88.89 to 97.56% and no errors on the test set, suggesting high relevance in forensic context.

Previous studies used the medial clavicle for age estimation almost exclusively by examining epiphysis union/non-union stages using several stages scoring system [5, 16–24]. In contrast, this research was not limited to any previously analyzed features directly but considered features of the images transformed into numeric variables (vector features). Although such an approach can be generally considered the black box, the interpretability in our case was assured through the domain-knowledge-based post-analysis of correctly/incorrectly classified bones. So, by visualization and field knowledge, we could relate processed images to age-related changes on the medial clavicle [33]. Initially, we proved the concept with unsupervised exploratory analysis, where we detected regularities in image grouping related to age; persons younger than 30 years were divided on one side of the graph, and the detailed analysis of their images showed incomplete fusion or fusion marks visibility. These morphological features align with the previous research [5, 16–19, 24, 25], which showed that fusion marks can be seen until 30. Also, some other morphological features were visible, such as surface flattening at age 40–60 and highly expressed porosity and bony nodules at age 80 [33]. The incorrectly classified samples were mainly those of people near the age limit of 30 years, an expected outcome considering the human body’s biological variability. Taking into consideration the forensic relevance of 30-year threshold in age estimation from medial clavicle, this study opted for that specific cut-off age although some age thresholds (e.g., 40 years) could have given better results from ML standpoint (as shown by PCA). Misclassifications were rare in the older age category as the morphological bone changes at that age are more straightforward and can hardly be misinterpreted as fusion marks. Some of the bones of younger people with visible union marks were classified as older adults due to the resemblance of the named changes with bony nodules, which are characteristics of the older age.

Although the results of this study in terms of methodology cannot directly be compared with the previous research that used the union and aging-related stages [62], the classification accuracies can be discussed. The study that used a five-phase scoring system also achieved a high PPV of 95.1% and specificity of 74.9% for the threshold of 18 years [62]. Similarly, the study on Magnetic Resonance Images (MRI) of clavicles achieved an accuracy of 88.5%. Most misclassifications were visible in the fourth and fifth stages, which could result from the lack of older individuals [61]. In our study, such issues were not relevant but overlapping, and consequently, incorrect classifications were common in individuals close to the classification threshold age. So, in our case, the age groups younger than 20 and those between 20 and 30 overlapped, probably because some younger individuals (ages 18 or 19) already exhibited bone flattening, but generally also because of the inherent biological variations in bone morphology where précised morphological threshold could not exist.

Although this research is a rare study that used deep learning and AI in anthropological biological profiling, the classification performance indicators were similar to previous research that used image analysis, such as those for sex estimation [34, 63, 64]. For example, the study using deep neural networks for sex estimation on the humerus achieved an accuracy of 91.03%, which proved to be higher than those of the forensic expert (83.33%) [34]. The other study employing neural networks achieved an accuracy on sex estimation of the skull 96.76% [64], and the study on teeth achieved an accuracy of 99.6% for sex and 93.8% for age estimation using a convolution neural network [65].

The interesting part of this study is the applicability of the Painters model, designated for the artwork assessment [44, 46]. Still, it was also applicable to age estimation by medial clavicle analysis, and this was one of the rare studies that used this model for any purpose other than the original one [66–68]. Although the selection of Painters model might not seem logical primary choice for such specific task, the preliminary assessment of most popular pre-trained networks (such as SqueezeNet [41], Inception v3 [42], VGG-16 [43], VGG-19 [43]) based on general image data set, showed lower performance than the model used in this study.

The application of deep neural networks in this study showed the potential for similar analysis in forensic anthropology, as the results indicate good classification power. The possibility of Orange to visualize the misclassified specimens minimized the effect of the black box approach. It enabled the involvement of forensic experts and pre-trained models to gain the best explanations of the model’s classifications. Future research could include other medial clavicle projections, as we used only the medial view, which could get even better results and lower the number of misclassified persons.

Although the results are promising, it should be stressed that the present study presents only proof of concept and the model at the prototype level. While the classification models achieved high accuracy, their interpretability may be limited, particularly with complex deep learning models. In addition to the field-knowledge interpretation that enabled explainability, an analysis of features, such as highlighting important regions of the image or visualizing pixel contributions, could minimize subjectivity, and strengthen the trustworthiness and usability of the developed models. This research relies on certain assumptions and methodological choices, such as the use of pre-trained neural networks and specific image analysis techniques. Alternative approaches or model architectures could yield different results and should be explored to assess the robustness of the proposed methodology. In the context of the ML applications, one of potential limitations is the sample size that could also limit generalizability of the findings. Additionally, as the study focused on a single population and geographic region, the applicability of the developed models to diverse demographic groups might not be the same.

The further steps should be full implementation of the model and development of the app that would enable direct application and additional validation of the method.

## Supporting information

S1 File Medial clavicles. (7Z)
